# Angiotensin-converting enzyme gene insertion/deletion polymorphism is associated with risk of oral precancerous lesion in betel quid chewers

**DOI:** 10.1038/sj.bjc.6602746

**Published:** 2005-09-05

**Authors:** F-M Chung, Y-H Yang, C-H Chen, C-C Lin, T-Y Shieh

**Affiliations:** 1 Department of Clinical Research, Pingtung Christian Hospital, Pingtung 900, Taiwan; 2 Graduate Institute of Dental Sciences, College of Dental Medicine, Kaohsiung Medical University, Kaohsiung, Taiwan; 3 Graduate Institute of Oral Health Sciences, College of Dental Medicine, Kaohsiung Medical University, No. 100, Shih-Chuan 1st Road, Kaohsiung 807, Taiwan; 4 Statistical Analysis Laboratory, Department of Clinical Research, Kaohsiung Medical University Chung-Ho Memorial Hospital, Kaohsiung 807, Taiwan

**Keywords:** betel quid, angiotensin-converting enzyme, oral precancerous lesions

## Abstract

To investigate whether *angiotensin-converting enzyme (ACE)* gene insertion/deletion (I/D) polymorphism is related to the risk of oral precancerous lesions (OPL) in Taiwanese subjects who chew betel quid, a total of 61 betel quid chewers having OPL were compared with 61 asymptomatic betel quid chewers matched for betel quid chewing duration and dosage. The frequency of homozygote for *ACE* D variant is significantly higher in the case subjects than that of the controls (44.3 *vs* 24.6%; *P*=0.0108). The adjusted odds ratio of the D homozygous for the risk of OPL is 8.10 (95% confidence interval (CI)=2.04–32.19, *P*=0.003). In the allelic base analysis, the D allele is also significantly associated with higher risk of OPL. When grouping the study subjects by smoking status, the association between *ACE* I/D polymorphism and risk of OPL was only observed in nonsmokers. Our results support the theory that genetic factors may contribute to the susceptibility of OPL and suggest that smoking and genetic factors may be differently involved in the development of OPL.

Both oral submucous fibrosis (OSF) and oral leukoplakia (OL) are chronic potentially malignant lesions of the oral cavity, the former being characterised by a submucosal fibrosis, resulting in marked rigidity and progressive difficulty in opening the mouth, the latter by chronic white mucosal maculae (Cox and Walker, 1996; Aziz, 1997). The most common oral premalignancies appear to be related to the habit of chewing betel quid, tobacco smoking and heavy alcohol consumption (Cox and Walker, 1996; Yang *et al*, 2001; Lee *et al*, 2003; Avon, 2004; Sikdar *et al*, 2004). These are chronic inflammatory and immunologic processes, which are believed to be attributable to the collective effect of genetic predisposing susceptibility in combination with specific environmental factors (Nair *et al*, 1999; Chiu *et al*, 2001, 2002; Kao *et al*, 2002; Liu *et al*, 2004; Shin *et al*, 2004; Sikdar *et al*, 2004; Tsai *et al*, 2004).

Angiotensin-converting enzyme (ACE) is expressed in a wide range of tissues (Smallridge *et al*, 1986; Noveral *et al*, 1987; Petrov *et al*, 2000) and converts angiotensin I to the potent vasoconstrictor angiotensin II (Ang II). The latter may be involved in the fibrotic process because it acts as a proinflammatory cytokine, participating in various steps of the inflammatory response (Mezzano *et al*, 2001). Studies have also demonstrated that *ACE* insertion/deletion (I/D) polymorphism is associated with fibrotic and atherosclerotic cardiovascular disease (Mezzano *et al*, 2001; Morrison *et al*, 2001; Arkwright *et al*, 2003; Luther *et al*, 2003). Thus, *ACE* might be a candidate gene for OSF and OL. We therefore investigated the role of *ACE gene* I/D polymorphism in relation to the risk of OSF and OL in the aboriginal population of Taiwan, which has a high prevalence of betel quid chewing.

## MATERIALS AND METHODS

### Recruitment of study subjects

Between January 2001 and December 2003, the Taiwan Bureau of National Health Insurance conducted the Taiwan Aboriginal Health Interview and Integrated Health Care Program in rural indigenous people over 30 years old. Using population registries, a total of 826 residents were screened for oral mucosal lesions in a Pai-wan tribe community in Pingtung County. Oral OSF and OL lesions were diagnosed on the basis of past history, oral examination and pathologic confirmation of the lesions according to the World Health Organisation (WHO) criteria (Wahi *et al*, 1966).

All study subjects lived in the same region at the time of the study and were of Pai-Wan ethnicity without known ancestry from other ethnicities. This study was approved by the Human Research Ethics Committee of our hospital, and informed consent was obtained from each participant. Each subject was interviewed face to face about demographic information, occupation, betel quid chewing, smoking history and alcohol drinking habits, as well as personal and family history of various cancers. The age at which the habit was started, average daily consumption quantity and the age at which the habit was stopped were queried in detail for betel quid chewing, cigarette smoking and alcohol drinking. Cumulative exposure to betel quid chewing was derived by multiplying the daily consumption of betel quid chewing (in quid/day) by the duration of betel quid chewing (in years). Valid questionnaires were examined further by a structured questionnaire. Blood pressure, body height and weight were measured and body mass index (BMI) was calculated. Blood was also collected; serum and white blood cells (WBCs) were separated on the day of blood collection and frozen at −70°C until subsequent analysis.

### Laboratory analysis

Genomic DNA was prepared from peripheral blood using standard techniques. For the ACE I/D polymorphism, the primer pairs used and the annealing temperatures were as follows: forward, 5′-CTGGAGACCACTCCCATCCTTTCT-3′; and reverse, 5′-GATGTGGCCATCACATTCGTCAGAT-3′, which amplify the intron 16 region where the I/D fragment is located. Polymerase chain reaction (PCR) amplification products were obtained using 25 *μ*l reactions (0.5 pg genomic DNA, 500 pmol of primers, 0.5 mmol l^−1^ each of deoxy-ATP, GTP, CTP, TTP and 1.5 mmol l^−1^ MgCl_2_; 0.5 U *Taq* DNA polymerase (Takara Tag™, Takara Shuzo Co., Ltd, Otsu Shiga, Japan); 50 mmol l^−1^ KCl; 0.001% gelatin; 10 mmol l^−1^ Tris-HCl, pH 8.3) with 4 min of denaturation at 94°C, followed by 35 cycles of 15 s at 94°C, 5 s at 67°C and 30 s at 74°C in a thermal cycler (Gene Amp PCR System 9700, Perkin-Elmer, Foster City, CA, USA). Reaction was terminated at 72°C at 2 min. To avoid ID/DD mistyping of heterozygote as DD homozygote (Shanmugam *et al*, 1993), all DD genotype samples were confirmed using a pair of primers that produce an amplified product only in the presence of the insertion, which was used to verify the polymorphism: forward, 5′-TGGGACCACAGCGCCCGCCACTAC-3′; and reverse, 5′-TCGCCAGCCCTCCCATGCCCATAA-3′ (Lee and Tsai, 2002). The PCR reaction condition was similar to the procedure for I/D detection, except that the annealing temperature was 62°C. All PCR products were visualised after electrophoresis on a 2% agarose gel and ethidium bromide staining. Genotyping was performed in a blinded manner.

### Statistical analyses

Data are shown as the mean±s.d. All statistical analyses were performed using SAS statistical software (Version 8.2, SAS Institute Inc., Cary, NC, USA). The two-sample *t*-tests or *χ*^2^ tests were used to compare means and proportions between the groups of control and oral precancerous lesions (OPL) when appropriate. To determine whether an association existed between *ACE* genotype and allele frequency with OPL risk, the significance of the difference in the distribution of genotypes and alleles between OPL patients and control subjects was calculated by *χ*^2^ statistic or Fisher's exact test and shown by *P*-value. All *P*-values were two-sided. A *P*-value <0.05 was considered to be statistically significant. Association of *ACE* genotypes and OPL was analysed by conditional logistic regression of OPL patients *vs* controls matched by betel quid chewing duration. To control the potential confounding effects, sex, age, BMI, blood pressure, smoking and drinking status were used as independent variables for adjustment. Conditional logistic regression was carried out with the SAS procedure PROC PHREG. Genotypic data entered the regression model in the form of two dummy variables representing the effect of genotype *ACE* I/D and genotype DD *vs* the reference category II, respectively.

## RESULTS

There were 185 subjects from a total of 826 rural indigenous people who were found on screening to have oral mucosal lesions and signed consent for further pathologic diagnosis. Among these, 111 subjects were confirmed to have OSF or OL; none had oral cancer, while a total of 50 subjects were excluded, 21 because of missing blood collection and 29 with missing information. Finally, a total of 61 persons were included as case subjects, 10 with OSF, 26 with OL and 25 OL with both lesions related to betel quid chewing. In all, 61 betel quid chewers without any previous or present lesions in the oral cavity matched for betel quid chewing duration and dosage were used as control subjects. Table 1 presents the clinical characteristics of study subjects. The mean age, gender distribution, BMI, blood pressure, amounts of quid consumption, drinking rate and duration, smoking rate and duration were similar in both groups. The proportions of betel quid chewing duration and cumulative betel quid consumption amount were also similar between the study groups.

The *ACE* gene I/D genotype distributions and allele frequencies of the study groups are presented in Table 2. The genotype distributions of this polymorphism were in Hardy–Weinberg equilibrium both in the case and control groups. The distribution of *ACE* genotypes in OPL patients was significantly different (*P*=0.0108) from that of controls. Patients with OPL had a higher distribution of DD genotype or D allele frequency, indicating the existence of a relationship between *ACE* gene polymorphism and OPL risk. The association between *ACE* DD and ID genotypes and risk of OPL still exist even after adjusting for age, gender, BMI, systolic blood pressure, smoking and drinking status by conditional logistic regression analysis (DD *vs* II: adjusted odds ratio (OR)=8.10, 95% confidence interval (CI)=2.04–32.19, *P*=0.0030; ID *vs* II: adjusted OR=3.69, 95% CI=0.98–13.81, *P*=0.0530). When analysis was performed comparing the risk of DD genotype *vs* II/ID genotype or ID/DD genotype *vs* II genotype and D allele *vs* I allele, DD genotype or D allele still had a significantly higher risk in the association with OPL (Table 2).

As previous reports have indicated that smoking status acts as a contributing factor for OPL (Cahan *et al*, 1976), we further subgrouped our study subjects according to their smoking status to examine whether if affects the impact of *ACE* I/D polymorphism with risk for OPL. The *ACE* gene I/D polymorphism was not statistically different between smoking betel quid chewers with or without OPL (Table 3), while the distribution of *ACE* genotypes in nonsmoking betel quid chewers with OPL was significantly different from that of those without OPL. Subjects with *ACE* DD and ID genotypes were significantly associated with the risk of OPL when adjusted for age, gender, BMI, blood pressure and drinking status (DD *vs* II: adjusted OR=14.62, 95% CI=2.47–86.44, *P*=0.0031; ID *vs* II: adjusted OR=6.97, 95% CI=1.23–39.46, *P*=0.0282). Further analysis comparing the risk of DD genotype *vs* II/ID genotype or ID/DD genotype *vs* II genotype and D allele *vs* I allele showed that DD genotype or D allele had a significantly higher risk in the association with OPL (Table 3).

## DISCUSSION

Although available epidemiological evidence indicates that the chewing of betel quid is an important risk for the development of OSF and OL (Avon, 2004; Yang *et al*, 2005), not all chewers develop oral mucosal lesions (Yang *et al*, 2001). Molecular epidemiologic studies have provided evidence that an individual's susceptibility to OSF and OL is modulated by both genetic and environmental factors. Inherited differences in the effectiveness of the disease may play a crucial role in host susceptibility (Chiu *et al*, 2002; Topcu *et al*, 2002).

Our previous studies confirmed that betel quid chewing acts as a major risk factor for OPL (Yang *et al*, 2001, 2005), while the present study further indicates that genetic factors exist in the vulnerability of betel quid chewing-related OPL. The additive used for betel quid chewing in Taiwan is quite different from that of India or Western populations. The betel quid in Taiwan does not contain any tobacco (Yang *et al*, 2005), while tobacco is usually added to betel quid chewed by Southeast Asians (Nandakumar *et al*, 1990; Sankaranarayanan *et al*, 1990). The present study shows that *ACE* gene I/D polymorphism is significantly associated with betel quid chewing-related OPL both in nonadjusted and adjusted models. To the best of our knowledge, this is the first report showing that *ACE* gene I/D polymorphism is associated with the risk of OPL in subjects who chew betel quid. Our results support the evidence that genetic factors may interact with environmental factors to contribute to the pathogenesis of OPL (Sikdar *et al*, 2004; Tsai *et al*, 2004).

Studies have suggested that molecular mimicry may explain the association among chronic inflammatory infiltrates, human leucocyte antigen and autoimmune disease with the pathogenesis of OSF and OL (Canniff *et al*, 1985; Tsai *et al*, 2004). Many cytokines have been thought to play important roles in the pathogenesis of OSF and OL (Chiu *et al*, 2001). Angiotensin II, the main peptide of the renin–angiotensin system (RAS), is a renal growth factor, inducing hyperplasia/hypertrophy depending on the cell type. It could also be involved in the fibrotic process of the oral cavity because of its behaviour as a proinflammatory cytokine, participating in various steps of the inflammatory response (Mezzano *et al*, 2001). Previous reports showed that subjects with DD genotype are associated with significantly higher levels of ACE and Ang II than those with I/I genotype, and individuals with the ID genotype have intermediate levels of these two enzymes.

Various immunologic changes are believed to play a role in the pathogenesis of OSF and OL (Canniff *et al*, 1985; Mega *et al*, 2001). The increase in CD4 complement and cells with HLA-DR in OSF tissues suggests that most lymphocytes are activated and that the number of Langerhans cells increases. Haque *et al* (1997) studied subjects with OSF with immunohistochemical methods and found immunocompetent cells to be present in the lesion, with a high ratio of CD4 to CD8 cells. Therefore, they suggested an ongoing cellular immune response leading to a possible imbalance of immunoregulation with eventual local alterations in tissue architecture. Angiotensin II is also known to play an important role in immune cells (Nakagawa *et al*, 2000), Papadopoulos *et al* (2000) showed that the *ACE* D allele in its homozygous form might confer susceptibility for autoimmune manifestations in sarcoidosis. Sato *et al* (1998) also revealed that the *ACE* genotype could be associated with the disease activity of SLE. Thus, subjects with the *ACE* gene D allele may, through the increased effect of Ang II, on cytokine activation, chronic inflammation and immune modulation, contribute to the increased risk of OSF and OL.

Cigarette smokers are more likely to develop many forms of disease than nonsmokers, including oral diseases. However, our study did not show an increased risk of precancerous lesions in smoking betel chewers with *ACE* gene D allele. Recently, Wang and co-workers reported that *GSTM1* deficiency is associated with the risk of vascular diseases, while they did not observe any interaction between the status of cigarette smoking and *GSTM1* deficiency in relation to coronary arterial disease severity (Wang and Wang, 2005). Similar findings were reported by Kelada *et al* (2000) that also did not find an interactive effect between smoking and *GSTM1 non-null* genotype of prostate cancer. Our results show that *ACE* gene I/D polymorphism and cigarette smoking status have no synergistic or additive effect in relation to the risk of OPL. This fact may suggest that smoking and genetic factors may be differently involved in the development of OPL disease.

In summary, our study shows an association of the *ACE* gene I/D polymorphism with OSF and OL in a Taiwan aboriginal population. These results imply that susceptibility to OSF and OL could involve genetic mechanisms modified by betel quid exposure. The present study also suggests that the RAS may be involved in the pathophysiology of inflammatory reaction and immunologic derangement in OSF and OL. Further work is required to confirm these findings in populations of different races and these preliminary exploratory results should be confirmed in a larger study.

## Figures and Tables

**Table 1:** Clinical parameters in patients with OPL and control subjects

**Parameters**	**Patients with OPL**	**Betel chewer control subjects**	***P*-value**
Age (years)	56.7±11.3	56.4±9.7	0.8839
Gender (male/female)	28/33	26/35	0.7154
BMI (kg/m^2^)	28.0±5.1	27.4±5.0	0.5336
Systolic BP (mmHg)	144.3±23.4	141.9±25.6	0.5991
Diastolic BP (mmHg)	86.8±13.0	83.1±14.0	0.1493

*Betel chewing duration (years)*			
1–20	16 (26.2)	16 (26.2)	1.0000
20–30	14 (23.0)	14 (23.0)	
30+	31 (50.8)	31 (50.8)	

*Cumulative amount of quid consumption*			
1–50 000	11 (18.6)	11 (19.3)	0.2923
50 000–100 000	7 (11.9)	14 (24.6)	
100 000–200 000	14 (23.7)	13 (22.8)	
200 000+	27 (45.8)	19 (33.3)	

*Alcohol drinking (%)*	45 (73.8)	41 (68.3)	0.5096
Drinking duration (years)	26.3±10.2	26.6±11.4	0.9040

*Smoking (%)*	17 (27.9)	19 (31.2)	0.6914
Smoking duration (years)	27.5±13.2	29.0±11.8	0.7190

OPL=oral precancerous lesion; BMI=body mass index; BP=blood pressure. Data are expressed as mean±s.d.; comparisons performed by unpaired *t*-test or *χ*^2^ test when appropriate.

**Table 2:** Comparison of *ACE* genotypes in patients studied

**Parameters**	**Patients with OPL**	**Control subjects**	***P*-value^a^**	**Crude OR**	**Adjusted OR^b^**	***P*-value**	**95% CI**
DD genotype (%)	27 (44.3)	15 (24.6)	0.0108	6.06	8.10	0.0030	2.04–32.19
ID genotype (%)	30 (49.2)	32 (52.5)		3.20	3.69	0.0530	0.98–13.81
II genotype (%)	4 (6.6)	14 (23.0)		1.00	1.00		

DD genotype (%)	27 (44.3)	15 (24.6)	0.0222	2.40	2.85	0.0127	1.25–6.51
II/ID genotype (%)	34 (55.7)	46 (75.4)		1.00	1.00		

ID/DD genotype (%)	57 (93.4)	47 (77.1)	0.0107	4.13	5.14	0.0115	1.44–18.32
II genotype (%)	4 (6.6)	14 (23.0)		1.00	1.00		

D allele (%)	84 (68.9)	62 (50.8)	0.0041	4.20	5.26	0.0003	2.13–13.00
I allele (%)	38 (31.2)	60 (49.2)		1.00	1.00		

*ACE*=angiotensin-converting enzyme; OPL=oral precancerous lesion; OR=odds ratio; CI=confidence intervals.

a Comparisons performed by *χ*^2^ tests.

b Adjusted by sex, age, body mass index, systolic blood pressure, smoking status and drinking status by conditional logistic regression analysis.

**Table 3:** Comparison of *ACE* I/D genotypes in patients studied grouped by smoking status

**Parameters**	**Patients with OPL**	**Control subjects**	***P*-value^a^**	**Crude OR**	**Adjusted OR^b^**	***P*-value**	**95% CI**
*Smoker*							
DD genotype (%)	6 (35.3)	5 (26.3)	0.8148	1.50	2.57	0.4932	0.17–38.03
ID genotype (%)	9 (52.9)	12 (63.2)		1.10	1.39	0.8129	0.09–21.81
II genotype (%)	2 (11.8)	2 (10.5)		1.00	1.00		
DD genotype (%)	6 (35.3)	5 (26.3)	0.5593	1.39	1.97	0.3955	0.41–9.45
II/ID genotype (%)	11 (64.7)	14 (73.7)		1.00	1.00		
ID/DD genotype (%)	15 (88.2)	17 (89.5)	0.9060	1.28	1.97	0.6080	0.15–26.24
II genotype (%)	2 (11.8)	2 (10.5)		1.00	1.00		
D allele (%)	21 (61.8)	22 (57.9)	0.7382	1.30	2.04	0.4563	0.31–13.31
I allele (%)	13 (38.2)	16 (42.1)		1.00	1.00		

*Nonsmoker*							
DD genotype (%)	21 (47.7)	10 (23.8)	0.0040	11.75	14.62	0.0031	2.47–86.44
ID genotype (%)	21 (47.7)	20 (47.6)		6.18	6.97	0.0282	1.23–39.46
II genotype (%)	2 (4.6)	12 (28.6)		1.00	1.00		
DD genotype (%)	21 (47.7)	10 (23.8)	0.0209	2.80	3.15	0.0225	1.18–8.42
II/ID genotype (%)	23 (52.3)	32 (76.2)		1.00	1.00		
ID/DD genotype (%)	42 (95.5)	30 (71.4)	0.0026	8.11	9.84	0.0074	1.85–52.51
II genotype (%)	2 (4.6)	12 (28.6)		1.00	1.00		
D allele (%)	63 (71.6)	40 (47.6)	0.0013	8.38	10.19	0.0001	3.09–33.59
I allele (%)	25 (28.4)	44 (52.4)		1.00	1.00		

*ACE* I/D=angiotensin-converting enzyme insertion/deletion; OPL=oral precancerous lesion; OR=odds ratio; CI=confidence intervals.

a Comparisons performed by Fisher’s exact test.

b Adjusted by sex, age, body mass index, systolic blood pressure, and drinking status by conditional logistic regression analysis.
